# Metallisation of Textiles and Protection of Conductive Layers: An Overview of Application Techniques

**DOI:** 10.3390/s21103508

**Published:** 2021-05-18

**Authors:** Alenka Ojstršek, Olivija Plohl, Selestina Gorgieva, Manja Kurečič, Urška Jančič, Silvo Hribernik, Darinka Fakin

**Affiliations:** 1 Faculty of Mechanical Engineering, University of Maribor, Smetanova 17, 2000 Maribor, Slovenia; olivija.plohl@um.si (O.P.); selestina.gorgieva@um.si (S.G.); manja.kurecic@um.si (M.K.); urska.jancic@um.si (U.J.); silvo.hribernik@um.si (S.H.); darinka.fakin@um.si (D.F.); 2 Faculty of Electrical Engineering and Computer Science, University of Maribor, Koroška cesta 46, 2000 Maribor, Slovenia

**Keywords:** metallization, conductive textiles, e-textiles, coatings techniques, protective coatings, durability

## Abstract

The rapid growth in wearable technology has recently stimulated the development of conductive textiles for broad application purposes, i.e., wearable electronics, heat generators, sensors, electromagnetic interference (EMI) shielding, optoelectronic and photonics. Textile material, which was always considered just as the interface between the wearer and the environment, now plays a more active role in different sectors, such as sport, healthcare, security, entertainment, military, and technical sectors, etc. This expansion in applied development of e-textiles is governed by a vast amount of research work conducted by increasingly interdisciplinary teams and presented systematic review highlights and assesses, in a comprehensive manner, recent research in the field of conductive textiles and their potential application for wearable electronics (so called e-textiles), as well as development of advanced application techniques to obtain conductivity, with emphasis on metal-containing coatings. Furthermore, an overview of protective compounds was provided, which are suitable for the protection of metallized textile surfaces against corrosion, mechanical forces, abrasion, and other external factors, influencing negatively on the adhesion and durability of the conductive layers during textiles’ lifetime (wear and care). The challenges, drawbacks and further opportunities in these fields are also discussed critically.

## 1. Introduction

The textile industry has adopted a forward-looking approach to create innovative conceptual textile systems for the 21st century globally, meeting the technological demands of modern society. Even in such a traditional area such as clothing, the modern lifestyle and new technologies are changing the aim and perception of textile materials, from simple protection to wearables with new functionalities and added value. Recently, researchers have shown an increased interest in flexible, electrically conductive textiles, which, in combination with different electronic components and circuit boards on the textile surface, represent electronic textile systems (so called e-textiles), with applications in photodynamic therapy, electronic sensors, flexible batteries, heating fabrics and light emitting displays [1,2,3]. Several approaches/techniques have been proposed in the literature about how conductivity can be implemented into flexible surfaces, (i) fabrics are weaved or knitted from conductive yarns, (ii) surfaces are sewn or embroidered with conductive threads, and (iii) those specially treated to impart conductivity, i.e., by chemical coating, surface metallization (e.g., copper (Cu), silver (Ag) or nickel (Ni) nanoparticles (NPs)), deposition of conductive fillers (carbon black or carbon nanotubes) and coating of conductive polymers (e.g., polyaniline, polypyrrole or poly-3,4-ethylenedioxythiophene), achieving large scale production of wearable e-textiles [4,5,6]. Moreover, the rapid development of nanoscience and nanotechnology has accelerated the miniaturization process of electronic devices [7]. In addition, e-textiles can have a wide field of actions, e.g., device-to device communications, cyber-physical systems, virtual/augmented reality, when coupled with solutions such as the Internet of things (IoT), data analysis (big data) or artificial intelligence (AI), among others [8].

This review introduces recent studies on the field of electrically conductive textiles obtained by thin layer of metal deposition onto the textile surface, so called metallization, their characteristics and diverse process parameters, and the most promising applications. Unlike previous reviews [5,9,10,11,12], the main contribution of this work focuses on frequently-mentioned metallization techniques with their advantages and drawbacks, i.e., printing of metallic inks, electroless deposition plating, electrochemical deposition, chemical vapor deposition (CVD), physical vapor deposition (PVD) and spray coating. The emphasis is also given on protective post-treatment coatings of conductive metallic layers to improve adhesion force and durability against washing, perspiration and abrasion, which is the key factor for the long-term functionality and performance of e-textiles. Closely connected to the usage of e-textiles is also a lack of unified and standardized procedures which would enable comparable testing and assessment in the production stage and even more importantly in the actual application phase; a designated section in the review paper is dedicated specifically to elaboration of these issues.

## 2. Conductive Textiles

Textiles themselves are considered as electrically non-conductive materials, since they do not conduct electric current. By the incorporation of conductive compounds into fibers/yarns during spinning or impregnation of a thin layer of conductive (nano)particles, fillers or polymers onto fibrous surfaces, using different techniques and procedures, textiles became electrically conductive. Some techniques, and their advantages and disadvantages, are reported and explained by different authors [3,5,6,13]. Due to structures’ heterogeneity and anisotropy, the electrical properties of conductive textiles depend on uniformity of the deposited conductive compounds, the arrangement of fibers and yarns in the textile, number of weft and warp yarns per length unit in weaved textiles, fabric thickness, elasticity and geometrical dimensions of the fabric, etc. [2,14,15]. Moreover, stretchability, shear bending, and other kinds of deformability of textile materials, as well unstable conductive coatings, also influence the electrical properties, enlarging sheet resistance [10]. Based on the final wearable application requirements, a low or high conductivity could be tailored, e.g., lower conductivity is preferred for heating applications and higher for lighting applications [16]. Whatever approach is used to attain conductivity, it does not have a negative impact on textiles´ weight, flexibility, and stretchability, whilst, from the ecologic and economic perspectives, it should remain nontoxic to humans and the environment, as well as cost effective. Moreover, the applied compounds should have good wash and wear durability, which remain key challenging properties.

Strictly speaking, conductive textiles are not “intelligent” or “smart”, because they do not react to outside stimuli (heat, chemicals, magnetism or mechanical stimuli), they are just primary components for enabling the creating of multifunctional electronically integrated textiles, or e-textiles [17], capable of sensing, heating, lighting or transmitting data, for diverse fields of applications, i.e., healthcare, military, sports and fitness, protective, automotive, entertainment and environmental applications [2,9,18,19]. They are also used for creating low profile switches in products where manufactured and hard conductive materials cannot be used. Fabrics can be cut, sewn, stretched, crumpled, and manipulated in ways where hard metals, carbon and plastics cannot be used. The global conductive textiles market size was estimated at USD 1.37 billion in 2018, growing at a CAGR of 16.5% from 2019 to 2025. Rising demands for the high-tech smart wearables from several end-use industries are likely to boost market demand in the forecast period.

Some examples of these novel products are flexible heaters, panels and actuators, electrostatic discharge clothing, high-performance sportswear and embedded health-monitoring devices (recording data related to body temperature, heartbeat, moisture, etc.), wearable pressure sensor (gait analysis and posture), flexible strain sensor for real-time human motion monitoring (detection and/or stimulate of muscle activity), portable power displays and light emitting transmission lines, EMI shielding textiles, textile antenna for wireless communication, tracking e-textiles (detect location and respond), interactive garments (changes in the garment activated by proximity, movement, light, projection, sound, voice, gaze and touch), and wearable electrodes for biopotential monitoring (cardiac, muscular, neural, and ocular biopotentials), etc. [2,3,5,10,19,20,21,22,23].

## 3. Fabrication Techniques for Tailoring of Conductive Textiles

The selection of fabrication technique for the production of conductive textiles is an important aspect in terms of process parameters that correlate with economic attractiveness, as well as affect the stability, flexibility, and physical-mechanical and electrical properties of the final e-textiles [7]. In general, there are two ways to manufacture conductive textiles. The first relies on the integration of conductive fibers, yarns, or threads into textiles by weaving, knitting, embroidering, etc. However, these processes are quite complex, and, furthermore, the integrated conductive fibers/yarns could influence the wear comfort of the fabric negatively, due to its rigidity and inflexibility [5]. The second approach is based on application of durable electrically conductive compounds (metals, conductive NPs, polymers, etc.) on the surface of textiles through different coating strategies, and, thus, realizing the electric properties in a finishing stage of the textiles. This eliminates the need to make the fibers electrically conductive during processing, and offers the possibility of coating the textiles with materials that cannot themselves form fibers, such as conductive polymers, that are not strong enough mechanically to pass through eventually harsh processes [24].

**Figure 1:**
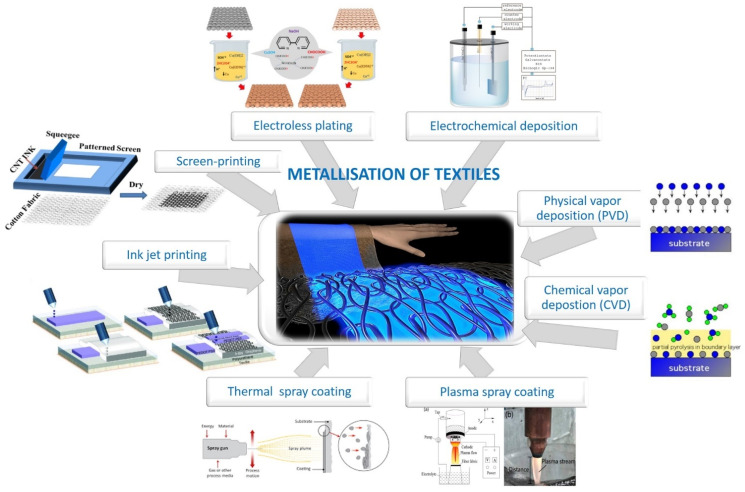
Schematic presentation of different metallization techniques to obtain electrically conductive textiles, namely, electroless plating (reprinted with permission from Ref. [6] Copyright 2021 Elsevier), electrochemical deposition (reprinted with permission from Ref. [27] Copyright 2021 Springer Nature), physical and chemical vapor deposition, thermal and plasma spray coating (reprinted with permission from Ref. [28] Copyright 2021 MDPI), inkjet (reprinted with permission from Ref. [29] Copyright 2021 Springer Nature), and screen-printing (adapted from Ref. [30]).

Among numerous methods to obtain conductivity, application of different metals on textile surfaces (so-called metallization) has received great attention, due to its uniqueness in providing multifunctional performance, i.e., electrical conductivity, EMI shielding, antibacterial and antifungal properties, etc. [6] or a shiny metallic appearance for decorative purposes [25]. The most commonly used methods for metallization of textiles can be divided into (Figure 1):

(i) Printing of metallic inks on the surface (inkjet, screen and stencil printing, lithography),
(ii) Deposition (electroless, electrochemical, physical vapor, chemical vapor),
(iii) Spray coating (thermal, cold, plasma-assisted).

The choice of appropriate methods depends on several parameters, such as operating temperature, type of metal, type and form of textile material, as well as final application, which is also associated with the desired thickness of the conductive layer [24].

The most frequently reported metals employed for conductive textiles are copper (Cu), silver (Ag), gold (Au), and nickel (Ni) [26]. Ag and Cu show the highest value of electrical conductivity (around 642 S/m), followed by Au (around 428 S/m). By taking the cost into account, Al and Cu possess the best conductivity/price ratio, around 2 and 0.9, respectively. Ni exhibits nine times lower conductivity/price ratio than Cu (around 0.1), but superior resistance to surface oxidation in air as compared to Cu. Low conductivity/price ratio can be observed for precious metals (Au, Ag, Pd, and Pt), and, therefore, they are less suitable for mass production.

### 3.1. Printing of Metallic Inks

Printing has several advantages over other techniques that provide electrical conductivity, including the local and direct deposition of conductive materials on various textile substrates at ambient conditions, obtaining of well-defined conductive patterns or images, the reduction in the consumption of conductive inks, the ability to transfer the entire process to the commercial manufacturing sector, possible large-scale production, and the significant reduction in costs associated with the production of e-textiles [7,22,31]. Considering the numerous conductive inks/pastes, special attention in this article will be paid to metallic inks, which are composed of metal (nano)particles (NPs), using different printing techniques such as inkjet printing, screen-printing and lithography.

Conductive inks or pastes should have certain properties, depending on the employed printing technique or the end use of the printed substrate. Generally, they are in the form of a dispersion/suspension of a conductive compound in a suitable solvent, with certain rheological properties. Conductive inks are usually formulated from three main components, the conductive compound, a binder that can aggregate the conductive particles efficiently, and a solvent that suspends the other components and gives the desired viscosity to the ink, regarding the chosen printing technique. The general requirements for inks or pastes are as follows; high electrical conductivity, easy to print, low-cost, good adhesion and stability on various substrates, resistant to oxidation, dry without clogging during printing, suitable surface tension and viscosity, and a low degree of particle aggregation [5]. Moreover, the biocompatibility of conductive inks for wearable electronics has become an important property in recent years [32,33]. On the other hand, metal inks and pastes are not durable to washing, and are susceptible to cracking and rubbing during usage, which need to be improved [6]. This is discussed further in detail in Section 4.

Recently, metal inks have been used extensively for the production of conductive coatings on flexible fibrous substrates, tailoring e-textiles [34]. The metallic (nano)particles in the ink solution/dispersion are usually stabilized by organic ligand shells. The (nano)particles are treated with an organic material such as polymers to prevent particle agglomeration and oxidation. However, after printing, the organic material should be removed by post-processing treatments, e.g., sintering, annealing or simple air drying, to allow physical contact between the metal particles to form a continuous path connection [32]. Due to the porous structure and rough surface of fibrous substrates, the uniform deposition of conductive (nano)ink is still a great challenge, leading to the inconsistency in electrical conductivity of the fabrics [30]. By far, the most commonly used metals present in metallic conductive inks are Ag [35,36,37], followed by Au [38,39,40]. In the case of Ag-based inks, the Ag oxidizes slowly on the surface and forms a thin conductive oxide layer that does not affect the ink‘s conductivity over time. Therefore, Ag-based inks are commercialized successfully by several companies (Henkel, DuPont, DycoTec, NovaCentrix, etc.), e.g., Intexar^TM^ fitness monitoring T-shirt with Wi-Fi connectivity (DuPont). On the other hand, high Ag loading is required for metal-based inks (more than 20 wt %) [41], so the main challenge is to find less expensive, but, at the same time, a highly-efficient conductive metal to replace the Ag (nano)particles. Aluminum (Al) [32] and Cu [34,42,43,44] have been proposed as alternatives to Ag, but they are not stable against oxidation, due to the formation of an insulating oxide layer that can affect the final conductivity of the ink greatly [32]. The advent of a new class of materials called MXenes has also been widening their application in printed electronics, overcoming the drawbacks of conductive inks such as poor conductivity [45].

#### 3.1.1. Screen Printing

**Figure 2:**
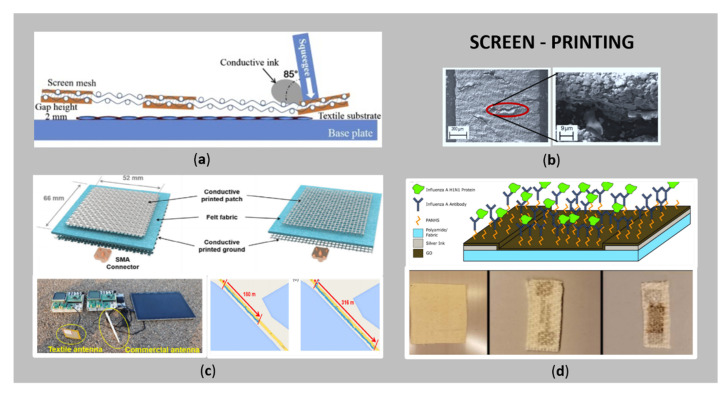
(**a**) Schematic diagram of screen printing in a cross-sectional view (reprinted with permission from Ref. [46] Copyright 2021 Elsevier); (**b**) a SEM image of printed Ag tracks on textiles (adapted from Ref. [47]); (**c**); schematic of textile patch antennas and their two-way Bluetooth communication system, using textile and commercial antennas (reprinted with permission from Ref. [48] Copyright 2021 John Wiley and Sons); (**d**) flexible screen-printed electrode on textile using Ag ink and a layer of graphene oxide (GO), and a schematic visualization cross-section showing the affinity assay for influenza (reprinted with permission from Ref. [35] Copyright 2021 IOP Publishing).

Figure 2a shows a schematic diagram of screen-printing in a cross-sectional view, as one of the most feasible, simple, reproducible, efficient, and low cost printing techniques for the tailoring of e-textiles [11,30].

The process is based on the printing of a conductive paste with suitable rheological properties (e.g., high viscosity) through a patterned fine mesh by means of a squeegee (different diameters and forms), that moves over the mesh with suitable speed and pressure, pressing the conductive paste onto the fabric surface. Thus, the desired conductive pattern is transferred to the substrate [22,26]. The use of the screen-printing process has several advantages: fewer printing steps are required to achieve conductive patterns, as this technique can deliver larger amounts of ink; the applied squeezing pressure improves the penetration and adhesion of the ink to the textile substrate, which improves the formation of interconnected conductive patterns; it is faster, and is already used as a technology in the industry for consumer products, e.g., wearable textile electrodes in physiological signal monitoring [22]. Since screen-printing is considered to have high processing efficiency, it is suitable for continuous roll-to-roll manufacturing, as has been pointed out by Jeong et al. [36] and Wang et al. [26]. The uniformity of the electrical conductivity of the printed pattern is affected significantly by the composition and viscosity of the conductive ink, the printing speed and number of printing layers, the distance between the screen mesh and the substrate, the physical and chemical characteristics of the substrate, and the mesh size [48]. In contrast, screen-printed patterns have a resolution of ten micrometres, which is slightly better than those produced by the direct-writing technique. Moreover, the inevitable drawback is the relatively thick layer of printed conductive pattern, causing rigidity of textiles, and, thus, this technique is less appropriate for flexible and stretchable e-textiles [26]. Figure 2b presents an example of scanning electron micrograph (SEM) images of Ag tracks on a polyurethane interface, with a corresponding SEM image at higher magnification showing the cracking of the conductive layer [47].

Figure 2c,d show the two latest application examples obtained by screen-printing of conductive pastes; i.e., Figure 2c textile patch antennas, which consist of a conductive layer printed on the flat and round yarn fabric and their two-way Bluetooth communication system comparing the textile and commercial antennas, with data transmission up to 316 m from the reference site (textile), and max transmission distance of 180 m (commercial) [48], and Figure 2d schematic affinity biosensor for detection of environmental exposure to influenza A virus for at-risk populations, fabricated using a flexible screen-printed electrode on the textile by combination of silver ink and a layer of graphene oxide (GO) [35]. Such kind of sensor, combined with IoT platform, could have the ability to predict potential influenza outbreaks before broad symptoms manifest.

#### 3.1.2. Inkjet Printing

**Figure 3:**
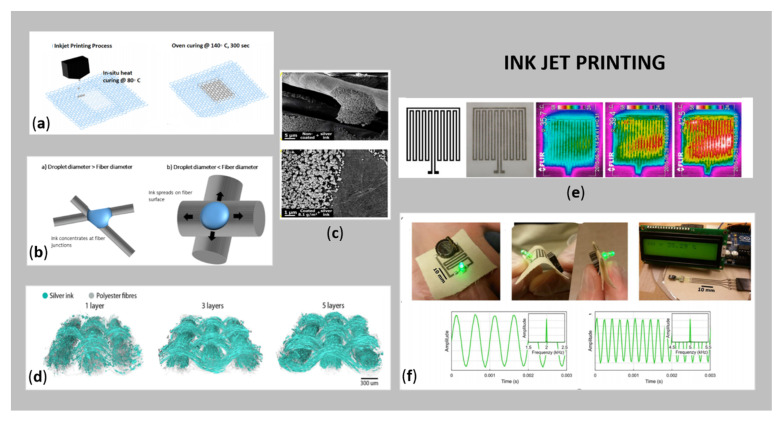
(**a**) Schematic presentation of inkjet printing of conductive Ag ink on textiles (reprinted with permission from Ref. [37] Copyright 2021, American Chemical Society); (**b**) presentation of a drop of Ag ink distribution on fibers with different diameters (reprinted with permission from Ref. [37] Copyright 2021, American Chemical Society); (**c**) SEM images of Ag inkjet printing, together with a combination of cellulose nanofibrils/glycerol on woven cotton fabric (reprinted with permission from Ref. [50] Copyright 2021, American Chemical Society); (**d**) XCT 3D reconstruction images of Ag (blue-green) deposited on polyester fabric (grey) with an increasing number of layers (reprinted with permission from Ref. [51] Copyright 2021 American Chemical Society); (**e**) textile heating actuators—computer designed patterns and thermal images of Ag inkjet printing PP spun non-woven textile (reprinted with permission from Ref. [52] Copyright 2021, Elsevier); and (**f**) digital moisture sensor signals recorded from the inkjet printing part on the fabric (reprinted with permission from Ref. [50] Copyright 2021, American Chemical Society).

The direct application of inks using a direct-writing device is called inkjet printing (nozzle movement controlled by software) and has recently been used widely for the manufacturing of conductive e-textiles (Figure 3). Herein, the patterned structures are fabricated dropwise using an electrically conductive ink by tuning the jet parameters or the properties of the conductive ink (Figure 3a,b). The process has become a very attractive alternative to conventional printing technologies (lithography, flexography and screen-printing), since no mask or screen is required, thus, the possibilities for changing the printing design are numerous. Furthermore, the conductive ink is usually consumed minimally, and the planned printed structure can be produced in a shorter time (of a few minutes) as compared to screen-printing. The formed final structures printed on substrates have high resolution, as well as the layer thickness is reduced, implying that this method is very appropriate for flexible and stretchable textiles. From an environmental point of view, the whole process does not require harsh chemicals and does not produce waste. On the other hand, for successful inkjet printing, the ink must contain the smallest possible electrically conductive NPs, and because of the oxidation problems of some metals, the stability of the ink suspension must be ensured by a protective layer on the conductive metal NPs. In addition, the use of highly viscous materials in the form of ink can lead to clogging of nozzles, which requires careful control of the rheological properties of the ink. The drying rate and wetting properties of the conductive metallic ink must also be considered. The use of metallic ink for inkjet printing is superior to other materials such as conductive polymers or carbon-based structures, due to their better conductivity [5,11,26]. However, the significant challenge for direct inkjet printing is printing accuracy and quality, due to ink droplets on the fabrics easily penetrating to the surroundings, which results in motion-blur patterns and poor conductivity. The printing accuracy is also affected greatly by the fabric structure, printing direction and ink properties [49].

Numerous researchers have used Ag-based inks in inkjet printing of wearable e-textiles [37,41,52,53,54,55,56]. Nechyporchuk et al. [50] reported inkjet printing of conductive circuits on fabric for a digital moisture sensor, using a combination of cellulose nanofibril-based coatings together with conductive Ag nanoparticle ink. SEM images are shown in Figure 3c, and photos of final samples together with sensor signals in Figure 3f. Li et al. [53] employed layer-by-layer inkjet printing of Ag NPs and GO film, together with Ni double hydroxide layers on flexible carbon fabric, resulting in a flexible and binder-free electrode for supercapacitors that exhibited excellent electrochemical performance. Krykpayev et al. 2017 [54] inkjet printed a wearable tracking device in the form of a complete localisation circuit with an integrated antenna on various fabrics, using commercial Ag-based ink. The best conductivity was observed on polyester/cotton 85%/15% fabric, up to 1.85 × 10^6^ S/m for six layers. Wang et al. [51] investigated the fluid-fibre interactions and corresponding conductivity of inkjet-printed Ag on a woven polyester (PES) substrate. The study, using X-ray tomographic reconstruction, showed that the distribution of inkjet-printed and sintered nanoparticulate conductive Ag ink depends strongly on the properties and architecture of the fibre surface (Figure 3d). Stempien et al. [52] deposited Ag patterns by inkjet printing on polypropylene (PP) nonwoven fabric for potential applications in textronic systems, e.g., capacitors, textile heating actuators (Figure 3e), textile patch antennas, and high-frequency transmission lines. In addition, Au-based metal electrodes have also been fabricated by inkjet printing on various fibrous substrates, as reported by [39,40], as well as Cu-based inkjet printing textiles [34,39,40,42,43].

#### 3.1.3. Lithography

Nano-imprinting lithography is the next promising technique for fabricating of conductive patterns at the nanometre scale. Typically, patterns are made by mechanical deformation of the imprint resist, followed by the post-treatment processes. During the fabrication of metal patterns, post deposition of selected metal on the already patterned layer is used commonly. In general, the metal can be deposited by various methods, e.g., direct metal evaporation, post-sintering, etc. [26]. Wang et al. [57] reported the continuous fabrication process of roll-to-roll ultraviolet nanoimprint lithography for the preparation of Ag mesh electrodes on polyethylene terephthalate (PET) substrate with a sheet resistance of 22.1 Ω/sq for flexible electronic application. Although nano-imprinting lithography enables high resolution of printed metal electrodes, the difficulties in manipulation and lack of processing efficiency limit its application in broader industrial use [26].

### 3.2. Electroless Plating

Electroless plating dates back to the 1940s [58]. The process was developed by Brenner and Riddell and patented in 1950. Thibodeaux and Baril in 1973 first reported this process as a means of metallising textiles to create them electrically and thermally conductive [59]. Electroless plating is a well-known controllable process, where either single metal NPs such as Cu, silver (Ag), nickel (Ni), gold (Au), platinum (Pt) and palladium (Pd), or a polyalloy like Ni-W, Cu-Ni, etc. are deposited chemically onto non-conductive (dielectric) substrates (printed circuit boards, glass, plastic, ceramics, and textiles) from an aqueous solution, without the use of an external electric current, in order to form a conductive, metallic layer [2,13]. Since many of these metals are autocatalytic, it is also called autocatalytic plating. Compared to other metallisation techniques employed on textile surfaces, i.e., in situ deposition of metal particles, flame and arc spraying, sputter coating and vacuum deposition, electroless plating has emerged as a superior method, due to its better performance regarding conductivity, durability, coherent metal deposition, industrial feasibility, relatively low cost, low temperature, no need vacuum (as is required for sputter coating), as well as applicability to complex-shaped materials [6,20,59,60]. Moreover, the process can be performed on different types (natural and synthetic-based) and forms of textile materials, i.e., fibres, yarns and flat textiles (knitted, woven or nonwoven). The overview of the latest studies is gathered in Table 1.

**Figure 4:**
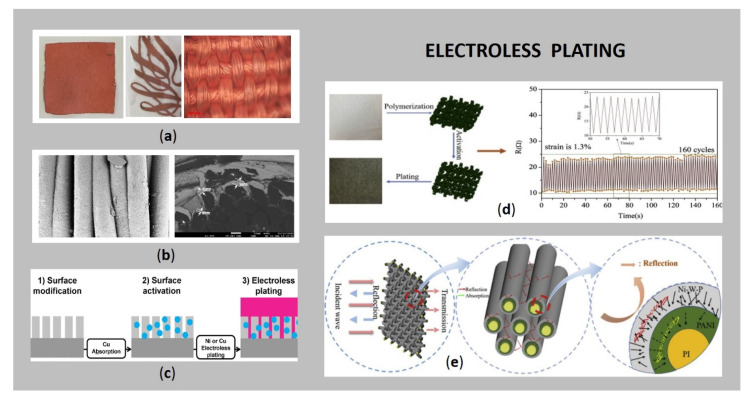
(**a**) Electroless Cu plated samples: the whole surface, pattern, and corresponding optical microscopy image; (**b**) SEM images of electroless Cu plated sample: longitudinal view and cross-sectional; (**c**) illustration of electroless plating process (reprinted with permission from Ref. [69] Copyright 2021, American Chemical Society); (**d**) sheet resistance during periodic stretching and releasing test at different strain levels, using a wearable strain sensor prepared by silver plated cotton/spandex blended fabric (reprinted with permission from Ref. [23] Copyright 2021, Elsevier); (**e**) the scheme of an EMI shielding mechanism of Ni-W-P/PANI/PI fabric (reprinted with permission from Ref. [61] Copyright 2021, Elsevier).

Flat textiles can be plated on the whole (large) surface area or, recently, as user-defined complex patterns (Figure 4a), utilised as an electronic circuit, pressure sensor, hydration sensor, heartbeat monitor, body temperature sensor, gait analysis and posture, for materials such as clothing, furniture and footwear, e.g., as used in assistive technology for older-adults [2,20,59].

As reported by Zhang et al. [70], highly conductive, well-defined patterns on a cotton textile can be produced by two consecutive steps, including non-diffusive Cu(II) patterning by a highly viscous Cu hydroxide solution and subsequent reducing-agent-dependent Cu deposition on textiles. Wills et al. [59] developed a selective patterning of Ag NPs to act as a catalyst (by a patent pending process) on cotton fabric using micro-dispensing and ink jet printing, followed by electroless Cu plating. Wu et al. [63] applied a stainless-steel stencil mask with the desired pattern on the top of the fabric, which provides patterned electrodes for light-emitting e-textiles (entertainment, fashion, and signalisation, etc.). Mao et al. [1] reported a well-defined Ag conductive pattern fabricated on PES fabric by screen printing of a dopamine surface modifier, followed by electroless Ag plating.

The conventional electroless plating method involves several chemical (cathodic and anodic) reactions on the same surface, where a source of metal ions is needed, as well as a reduction agent, a complexing agent, a stabilizer, a buffering agent and a wetting agent, regarding the type of plated metal [2,24]. Thus, the process is composed of three related phases (Figure 4c): (i) pre-treatment (surface modification), (ii) a catalytic process (sensitising, activating, and accelerating), and (iii) electroless plating [71], followed by (intermediate) rinsing and drying.

To prepare the surface for subsequent metal deposition, pre-cleaning of textiles is usually carried out in aqueous solution, typically at a liquor to material ratio of 1:50, using non-ionic detergent, at temperatures of 40 up to 95 °C, for 10 up to 40 min [2,61,65,72], depending on the type of material, since various additives and contaminants on a textile surface can influence the metal deposition and operational conditions negatively. As an alternative to washing, ultrasonication was used in different alcohol solutions for 10–20 min [13,63,64,73]. Some researchers pre-treated textiles in an alkaline solution at high temperature, e.g., flax fabric [13], PES fabric [71], cotton/polyurethane (Co/PU) 95/5 [23], or exposed the surface to plasma treatment for a shorter time, e.g., Co fabric [64], polyamide/polyurethane (PA/PU) 87/13 [63], or laser treatment, e.g., Co fabric [74]. Moreover, to improve the binding ability between the metallic functional layer and the textile substrate, the matrix material can be modified with a coupling agent such as silane-based compounds (3-aminopropyltrimethoxysilane (APTMS) and 3-mercaptopropyltrimethoxysilane (MPTMS)) [75], dopamine [64,76], chitosan, cationic polymer Poly(diallyldimethylammonium chloride) (polyDADMAC) [59], aniline monomers [61], etc., although the APTMS and MPTMS are harmful to skin when contacting with the human body, which limits their application for wearables [13].

The process of electroless plating of non-conductive materials proceeds by sensitising the surface of a substrate with a catalyst, with the aim to initiate metal deposition at these catalyst sites by enhancing the rate of reduction and uniformity of the plating metal ions [59], thus, the choice of an efficient catalyst is critical. This step is usually quite expensive, since it requires the application of noble metal ions such as Pd and Au [60]. As mentioned by Lu et al. [73], Pd/Sn colloid is the most widely used catalyst, where the Pd chemisorption can be carried-out either by a two-step method, i.e., the substrate to be coated is firstly immersed into tin(II) chloride, and, after that, in palladium, or (II) a one-step process, i.e., PdCl_2_-SnCl_2_ colloidal solution is used [77]. Because of high cost of Pd and its presence on the critical raw materials list in Europe, Pd-free catalysts are desirable. Ag [20,62,64], and Cu [20] could be alternative candidates (Table 1), although Cu is more favourable due to being earth abundant, and its stability and significantly lower cost. In a recent paper, Azar et al. [20], reported a thin and non-diffusive coating of a highly viscous Cu(II) solution applied to the textile material in a first step. Although Cu is less active than Pd or Ag, its performance can be enhanced by functionalization of Cu NPs with different ligands, such as adipic acid, silanes and octadecanethiol with two functional groups (i.e., carboxylic acid, methyl or amine groups), improving electroless Cu plating. Recently, different authors have studied electroless plating processes without addition of stabilising and sensitising agents. Thus, Moazzenchi and Montazer [78] reported a simple click electroless Cu plating and sonoplating of polyester fabric for E-textile applications; Karami et al. [65] electroless Ag plated surface of reduced GO (rGO) coated cotton fabric for textile-based super-capacitor electrode applications; Takuoka et al. [66] electroless Ni-P plated PET textile with the assistance of supercritical CO_2_ (scCO_2_); Ali et al. [6] focused on metallization of cotton fabrics by using the shorter route of modified electroless Cu plating method, which is also Pd, Sn and formaldehyde free; Lu et al. [73] studied an efficient catalyst-free process for the electroless Cu plated bamboo fabric initially-modified with MPTMS ethanol solution.

The reducing agent is needed for the transformation of metal ions in the electrolyte solution to metallic atoms on the fibrous surface [6,24]. This catalytic redox reaction takes place in alkaline medium at high temperatures, resulting in a uniform metallic coating wrapping the entire surface of an individual fibre, as could be seen from the SEM images (Figure 4b). The most commonly employed reduction agent during Cu electroless deposition is formaldehyde, due to its low cost and effectiveness, as well as ease of control of formaldehyde systems [6,79]. Because of the formation of hazardous gaseous products during the plating process and its toxicity, there is an increasing demand for using formaldehyde-free reducing agents. Herein, different authors reported amine-borane, dimethylamine-borane [73], trimethylamine-borane [80], sodium borohydride [13,59], sodium hypophosphite, [79], a combination of sodium borohydride and sodium hypophosphite [62], sodium hydrosulphite [6], hydrazine hydrate [72], glyoxylic acid [79], Cu(II), Fe(II) [79], glucose [64,68], etc., as efficient substitutes for formaldehyde. In order to prevent metal precipitation due to pH changes and to enable operating at higher pH, numerous complexing agents (e.g., ethylenediaminetetraacetic acid (EDTA), sodium citrate, malic acid, lactic acid, glycolic acid, etc.) have been explored [81,82]. In addition, a stabilizer (e.g., thiourea) is added (1–100 ppm) to increase bath stability and prevent random deposition of the metals in a solution [83]. Furthermore, polyethene glycol and K_4_Fe(CN)_6_ are reported to improve resistivity after Cu plating [24]. Other parameters, like pH, plating temperature, surface quality and thickness, also influence the conductivity of plated textiles.

As can be seen from Table 1, the most commonly electroless deposited metal on different textiles‘ surfaces is Cu, followed by Ag and Ni. Less popular for metallization of textiles, although quite often applied as catalysts, are noble metals such as Pt, Pd and Au, due to their high price. Electroless deposition of Cu is very popular in printed circuit board (PCB) manufacturing, and in recent years, for textile metallisation, offering several advantages before other techniques, such as high selectivity, industrial feasibility, high conductivity, uniform plating thickness, uniform physical and mechanical properties, the ability to create complex patterns, deposition on non-conducting surface, high process throughput, low costs of tools and materials [6,20,24,59]. On the other hand, it is limited due to Cu‘s low oxidation resistance, which could be enhanced by application of protective compounds (as described in Section 4) and low electromagnetic shielding performance [2,61]. The most widely used Cu deposition solution is composed of cupric salt (Cu sulphate, chloride, or nitrate), formaldehyde, sodium hydroxide, EDTA and additives in well-defined concentrations. The optimal pH of formaldehyde containing electroless Cu plating baths is around 12.5. Among metals, Ag provides the highest conductivity, and is one of the most preferred materials due to its biocompatibility, stability, and antibacterial property [22,23]. Before electroless Ag deposition, it is very important to modify the textile surface, to improve the binding ability between the Ag particles and the substrate [76]. As already mentioned, this could be achieved using dopamine (or amine), which transforms over self-polymerisation into polydopamine (or polyimine), which contains many functional groups on its surface responsible for bonding. The electroless Ag plating fabric has a stronger shielding effect compared to Cu and Ni deposition, because of its higher electrical conductivity [61]. Because of its good antibacterial properties, Ag can be used for a strain sensor for human motion, as well as the direction of human motion monitoring (detection and/or stimulate of muscle activity, robotic skin, assistive technology, etc.), as reported by Ma et al. [23] (Figure 4d). In addition, the electroless Ni plating has been used in many applications because of its deposit properties, including good electrical and magnetic properties, excellent corrosion and wear resistance, and deposit uniformity [77], with the drawback of low conductivity and electromagnetic shielding performance [61]. The electroless Ni bath typically comprises an aqueous solution of nickel sulphate (source of Ni), complexing agents and hypophosphate (reducing agent), operating in a specific temperature and in a specific pH range to provide bath stability; but the generation of by-products lowers the pH, thus, alkaline salts of Na and K are usually added [84]. Since it could cause allergic contact dermatitis, Ni is the least appropriate metal for applications that are in close contact with the skin. In order to improve the properties of single metal electroless deposition, e.g., conductivity, oxidation resistance, shielding ability, hardness or anti-nuclear radiation properties, different (poly)alloys like Ni-W, Cu-Ni [13] or ternary Ni-X-P coatings have been developed, where X is typically a transition metal such as Co, Cu, Mo and W [61]. Figure 4e shows the scheme of the EMI shielding mechanism of Ni-W-P/PANI/PI fabric.

### 3.3. Electrochemical Deposition

Electrochemical deposition (or electroplating) of a thin layer of reduced metal ions on electrically conductive substrates using electric current leads to surfaces´ metal coating (metallisation) [11]. Before plating, the substrate must be cleaned before electroplating to remove impurities, resulting in effective attachment of the metal particles to the surface. The process is affected by various parameters, such as the coating-environment interface, the coating-substrate interface and the coating material itself [22,26,85]. In general, three main phases can be identified in cathodic metal deposition: (i) Ion migration, (ii) electron transfer, and (iii) intercalation of adsorbed metal ions [86].

**Figure 5:**
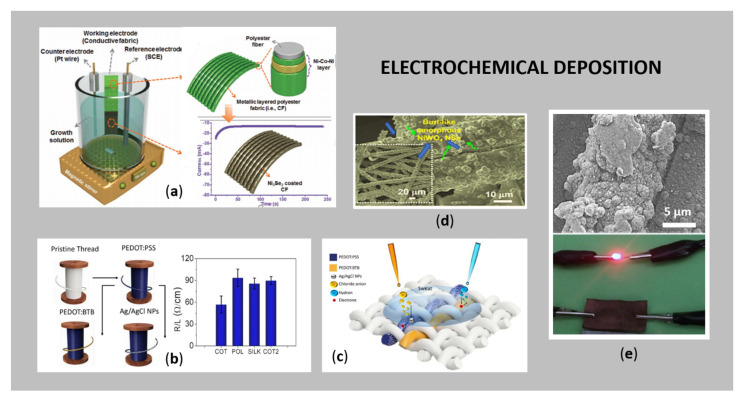
(**a**) Schematic electrodeposition process with a three-electrode system (reprinted with permission from Ref. [98] Copyright 2021, John Wiley and Sons); (**b**) average resistance values of differently electroplated conductive threads (reprinted with permission from Ref. [97] Copyright 2021, Springer Nature Limited); (**c**) working principle of the thread sensor that detects chloride ions, pH and sweat simultaneously (reprinted with permission from Ref. [97] Copyright 2021, Springer Nature Limited); (**d**) SEM image of PET coated uniformly with amorphous NiWO_4_ (adapted from Ref. [89]); (**e**) SEM of Cu NP-coated PET knitted fabric and corresponding conductivity test (adapted from Ref. [99]).

The electrochemical deposition of the conductive films on a flexible substrate has several advantages over other techniques, such as controlling the morphology and thickness by adjusting the deposition parameters (e.g., electrolyte concentration and composition, deposition time, density of the applied current). Moreover, the process requires low-cost equipment for an economically affordable process, and the process is simple with a high deposition rate [26]. The major drawback using cathodic metal deposition is that the substrate intended for coating should be electrically conductive, otherwise the current cannot flow. Since textiles usually act as natural insulators, it is necessary for them to become conductive prior to electrodeposition. This is feasible by the creation of conductive layers (in a pre-treatment step) on a textile surface, using different metals, such as Ag [51,87], Cu, [88,89,90], Ni [91], Mxenes [92], Ni-Co selenide nanowires [93], NiMoO_4_/CoMoO_4_ nanorods [94], LaMnO_3_/MnO nanoarrays, ZnO nanostructures deposited on an Ni-Cu-Ni conductive layer [95], and LiMn_2_O_4_ [96], conductive polymers [97] or carbon-based materials [11,22,23,24,25,26]. Schematic presentation of a typical electrochemical deposition process of a metallic layer onto PES fabric, using a three-electrode system, is shown in Figure 5a, where the deposition of Ni_3_Se_2_ NPs is carried out on the conductive fabric (used as a working electrode), resulting in a metallic polyester layered fabric. The chronoamperometry curve suddenly raised during the electrochemical process, and, after that, became stable [98].

Figure 5b shows the average resistance values of different threads, pre-treated with PEDOT:PSS and PEDOT:BTB conductive polymers, and afterwards electrodeposited with Ag/AgCl NPs. In this way, the constructed thread sensors were used for the simultaneous detection of pH, sweat and chloride ions, as can be seen from Figure 5c. Nagaraju et al. [88] electrochemically deposited self-assembled hierarchical β-cobalt hydroxide nanostructures on Cu pre-coated PET fibres, which were further used successfully for supercapacitors. In another study, Nagaraju et al. 2015 [89] electrodeposited amorphous NiWO_4_ nanostructures on Cu pre-coated PET fibres. The as formed metal coating was examined in detail using SEM analysis (Figure 5d), revealing that the conductive Cu-coated PET was covered uniformly with NiWO_4_ nanostructures.

Among the aforementioned metals, Cu is an excellent option for pre-treatment of textile substrates, as it usually covers minor imperfections. In the case of electrodeposition, it is relatively inert, low-cost, has high deposition efficiency and is highly conductive [24]. Li et al. [100] employed Cu-polythiophene composite film as a seed layer for direct electrodeposition, resulting in a stretchable fabric with highly stable electrical conductivity for monitoring personal health. Similarly, in situ deposition of Cu particles on cotton fabric was studied by Ali et al. [101] for its applicability in EMI shielding. They found out that fabrics with a dense, uniform, and percolated network of conductive Cu particles on the surface, which were produced from 100 and 150 dips, exhibited the maximum shielding ability of 10 dB and 13 dB. Zhao et al. [102] prepared flexible polyaniline/polyethylene terephthalate (PANI/PET) conductive textiles by chemical polymerisation of PANI on PET textiles, and, subsequently, electrochemical deposition of Cu crystals for possible e-textiles´ applications. Recently, a novel technology, the so-called hydrogen evolution-assisted electroplating, was used for the deposition of Cu, improving deposition rates as compared to the conventional electroplating method [103]. Cheng et al. [99] reported polydopamine pre-treated PET knitted fabric as a template for in situ hydrothermal growth of Cu NPs, with superb stability of resistance-strain behaviour in the long-time cycle test (Figure 5e). The designed system demonstrated its applicability as wearable strain sensors, i.e., detect real-time motion of biceps brachii, monitor the walking of shank, detect, subtle deformation of human body, detect the muscle movements during swallowing, etc., as well as electric heaters, i.e., for thermal therapy on hand.

### 3.4. Vapour Deposition Techniques

Vapour deposition refers to the atomistic deposition process where the coating material is vaporised and condensed onto the selected substrate in a vacuum, forming a thin film (~10 μm for physical deposition and ~1000 μm for chemical deposition) from virtually all types of inorganic (metals, alloys, compounds, and mixtures) and some organic materials [104]. Herein, two fundamental solvent-free processes that utilise vacuum deposition are known as chemical vapour deposition (CVD), where the reaction between volatile precursors and the surface of the coated materials occurs, and physical vapour deposition (PVD), where evaporation and sputtering occur. Both CVD and PVD have opened new possibilities in the modification of textile materials, and they are an exciting prospect for use as e-textiles. CVD is just described briefly below, since it has not been studied recently in articles as a technique for metallisation of textiles to obtain their conductivity.

#### 3.4.1. Chemical Vapor Deposition (CVD)

CVD is an umbrella term for various deposition techniques, i.e., atmospheric pressure chemical vapour deposition, organometallic chemical vapour deposition, low pressure chemical vapour deposition, laser chemical vapour deposition, photochemical vapour deposition, chemical vapour infiltration, jet chemical epitaxy and plasma-enhanced chemical vapour deposition. The process steps in CVD include transport of precursor molecules into the reactor, diffusion of precursor molecules to the surface, adsorption of precursor molecules on the surface, reactions on the surface: decomposition of precursor molecules on the surface and incorporation into a solid layer, and recombination of molecular by-products and desorption into the gas phase [105]. When the precursor gases flow over the surface of the heated substrate, the resulting chemical reaction forms a solid phase that is deposited on the substrate, and the temperature can influence the reaction process significantly.

#### 3.4.2. Physical Vapor Deposition (PVD)

PVD has many advantages over other deposition techniques (also over CVD), including high purity, efficiency and environmental friendliness, use of substrates in the form of mainly pure gases and metals instead of their expensive, complex and usually toxic chemical compounds; the possibility of producing composite coatings (through multiplex technology) in multi-source devices; unlimited possibilities in the field of production of coating materials due to free adaptation of the substrates, and, thus, the wide possibilities in terms of designing various functional properties of coatings, e.g., chemical, mechanical, tribological, physical, thermal, anticorrosive, etc., the possibility of producing both non-stoichiometric and non-equilibrium coating materials with different properties [106]. The major drawback of the PVD method is insufficient adhesion of the deposited layers, due to the non-homogeneity of the textile surfaces, which can result in a lack of electrical conductivity at larger areas [107]. Several types of PVD are used for the modification of textiles, i.e., cathodic arc deposition, physical vapour deposition with electron beam, evaporative deposition, pulsed laser deposition and sputter deposition [106].

**Figure 6:**
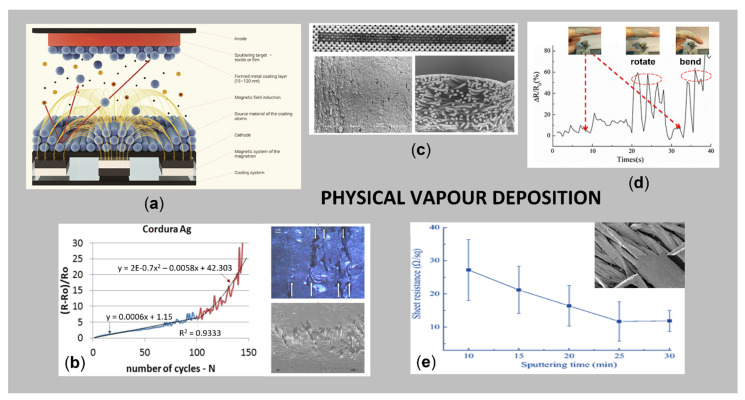
(**a**) Basic mechanism of the magnetron sputtering process; (**b**) relative change of resistance of Ag coated Cordura subjected to cyclic bending, and corresponding microscopic and SEM images after 10,000 bending cycles (reprinted with permission from Ref. [107] Copyright 2021, Springer); (**c**) magnetron sputtered Cu transmission lines on polypropylene nonwoven and responsible SEM images: longitudinal view and cross-section (reprinted with permission from Ref. [110] Copyright 2021, Łukasiewicz Research Network—Institute of Biopolymers and Chemical Fibres); (**d**) monitoring the finger motions by Ag/G-coated cotton: rotation and bending (adapted from Ref. [111]); (**e**) sheet resistance of Ag/polypyrrole-coated cotton as a function of sputtering time and corresponding SEM at 10 min (adapted from Ref. [115]).

Among the above-mentioned variations, the magnetron sputtering is the most frequently employed process for the deposition of thin, durable layer of metals (Cu, Ti, Ag, Al, W, Ni, Sn, Pt), metal oxides (TiO_2_, Fe_2_O_3_, WO_3_, ZnO) or non-metallic compounds (Si, graphite, ceramic) on various types and forms of flat textiles [108], providing a sophisticated film thickness and user-defined patterns on a substrate [109] for diverse applications, i.e., electroconductive transmission lines for emergency and security services [110], flexible strain sensors for real-time monitoring of human motions [111], EMI shielding protective gear [112,113], etc. Moreover, the transfer from laboratory results to industrial applications is rather straightforward, since magnetron sources can be upscaled easily [114]. The basic mechanism of the magnetron sputtering process is presented in Figure 6a.

Nowak et al. [110] magnetron sputtered Cu electroconductive transmission lines on PP and PA nonwovens by varying the sputtering time and layer thickness, for potential applications in clothing for the emergency and security services (Figure 6c). The results have shown that it is possible to obtain a surface resistivity of approx. 0.2 Ω on spun-bonded PP nonwoven, at a sputtering time of 180 min and Cu layer thickness of 2.2 µm. In the work of Huang et al. [113], the nano-Cu film was deposited on the surface of polyester fabric, and the structure and porosity dependent EM shielding capacitance, electrical conductivity and UV protection were presented. Chu et al. [116] developed easily processable and robust conductive PP fibres appropriate for many fields, such as antibacterial resistance, Joule heating, and electromagnetic shielding, using the magnetron sputtering technique with pure Cu and Ag targets in the presence of Ar gas. Even though magnetron sputtering technology has already demonstrated its usefulness in conferring EM shielding and electrical conductivity of materials, the problems related to the stability of the metal deposition in terms of human safety need to be investigated further [108].

Rani et al. [117] reported a successful plasma assisted sputtering process of Cu on polyester/silk blended fabrics for preparation of multifunctional properties, where the effects of power, time, and the gas flow have been examined. Meng et al. [118] studied the influence of the atmospheric environment on the conductivity of deposited Cu films on polyester substrate by RF (radio frequency) magnetron sputtering and low-temperature plasma technology. The obtained results showed that Cu films were broken, and the continuity of samples was destroyed after 60 days, but the conductivity of the samples had hardly changed with the ambient temperature, humidity and degree of water washing, which is decided mainly by the internal structures of substrates. Korzeniewska et al. [107] studied the mechanical durability of a vacuum deposited Au and Ag layer on an elastic composite material Cordura^®^ (nylon coated with PU film) and PTFE membrane by describing the mathematical changes in the resistance of structures depending on the number of bending cycles (Figure 6b).

In addition, highly conductive textiles are prepared using different two-step processes that combined pre-treatment and magnetron sputtering. He et al. [115] endowed cotton fabric with high electrical conductivity employing in situ polymerisation of PPy, and, after that, Ag thin film, by direct current (DC) magnetron sputtering (Figure 6e). The resultant Ag/PPy-coated cotton deposited with a sputtering power of 200 W for 25 min had the highest electrical conductivity, with an average sheet resistance of 11.7 Ω/sq. Moreover, it has high hydrophobicity, thermal stability, electromechanical performance and washing fastness. In another study, He et al. 2019 [111] modified cotton with a continuous rGO thin film using a dipping method, and then coated it with Ag thin films using a magnetron sputtering system, with the aim to fabricate a strain sensor, obtaining average surface resistance of 2.71 Ω/sq. The as prepared strain sensor for real-time monitoring of human motions has the advantages of high sensitivity, a large workable strain range (0–20%), fast response and great stability (Figure 6d). Jiang et al. [119] pre-treated cotton fabric with polyvinyl alcohol (PVA) to reduce the porosity of the fabric and then Cu or Ti was sputtered, resulting in improved electrical conductivity and EMI shielding ability. Depla et al. [120] reported pre-treatment of woven and nonwoven substrates in a glow discharge, and subsequent magnetron sputtering of metal thin films (Al, Cu and Ti) and oxide thin films (Al_2_O_3_, TiO_x_) for a smart textile, that is, a thermocouple deposited on a woven PP substrate, obtaining good adhesion between textiles and coatings.

Sedighi et al. [112] sputtered Ag NPs on one side and GO was sprayed on the other side of a nylon/PES fabric, followed by chemical reduction, in order to use the as prepared composites as flexible electrodes in wearable electronics. In addition, the durability of the modified fabric was investigated under mechanical deformation, as well as its antibacterial activity. Liu et al. [121] deposited a Ti/Ag/Ti tri-layer and Ti–Ag alloy thin films on the surfaces of PET, which exhibited excellent electrical property with electrical resistivity of 5.1 × 10^−7^ Ω/m and 3.4 × 10^−7^ Ω/m, respectively. Moreover, the samples retained ideal flexibility and adhesion.

### 3.5. Spray Coating

**Figure 7:**
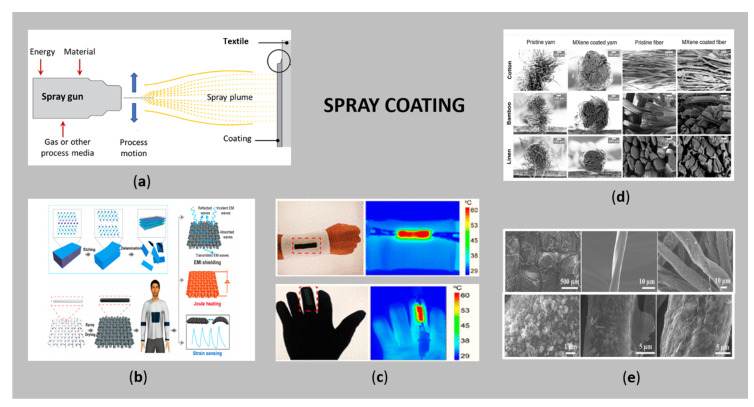
(**a**) Schematic of the thermal spraying (adapted from Ref. [122]); (**b**) scheme of the spray coating process of previously fabricated MXene to attain multifunctional electrically conductive cotton fabric (reprinted with permission from Ref. [127] Copyright 2021, American Chemical Society); (**c**) temperature distribution of the wearable heater attached on a wrist and glove (adapted from Ref. [127]); (**d**) SEM morphology of different cellulose yarns spray coated with the conductive Mxene flakes (reprinted with permission from Ref. [128] Copyright 2021, John Wiley and Sons); (**e**) SEM of ZnO and GO-coated cotton fabric using different spray coating cycles for EMI shielding (reprinted with permission from Ref. [129] Copyright 2021, Elsevier).

Thermal spraying (Figure 7a) is an emerging and promising coating technique, where melted or heated (conductive) compounds are sprayed onto different surfaces in the frame of droplets under air pressure, where they solidifiy and bond upon contact, forming a stable and hard-wearing coating [122]. The “feedstock” (coating precursor) is heated by electrical (plasma or arc) or chemical means (combustion flame). This technique provides thick coatings (in the range of 20 µm to several mm, depending on the process and feedstock), on a larger surface area and with a higher deposition rate, independently on surface morphology, as compared to other coating processes such as electroplating, PVD and CVD. Moreover, a screen mesh or shadow mask can be used, thus, the sprayed components can be distributed in the desired locations and in the form of custom-designed patterns [123,124,125]. For these reasons, spray coating has recently gained great interest for the fabrication of versatile conductive wearable textiles. In addition to the attractiveness of the process, special attention must be paid to the drying rate, control of rheological properties of the feedstock and wetting properties of the textiles. Precise control of the deposition is also questionable, which limits its wide applicability [26]. Moreover, the process involves the melting of the sprayed particles, which can damage heat-sensitive substrates [126].

Voyer [125] used the wire flame spray process to fabricate electrically conductive Al coatings onto diverse textile fabrics, which were further used as cathodes in flexible Li-ion batteries. He found out that sufficient electrical conductivity could be achieved by a coating quantity threshold of about 200 g/m^2^, and an excellent conductivity (about 500 S/m) through an adequate optimisation of the spraying parameters. On the other hand, cold spray is very attractive for the achievement of metallisation of low-temperature resistant materials such as organic composites, due to its “cold” characteristic. Giraud et al. [126] demonstrated the feasibility of cold spray for satisfactory metallization of PA66-matrix composites with Al, studying the influence of the processing parameters such as carrier gas temperature and pressure.

Recently, ultrasonic spraying has been shown as an excellent alternative to support conventional spray coating, where the use of ultrasonic frequencies can easily control droplet size, resulting in more homogeneous dispersions being sprayed (the vibrations‘ break up potentially formed particle clusters) and an improved uniform conductive layer [124]. Furthermore, supersonic cold spraying (a non-vacuum, binder-free, fast, scalable process) has proven to be very suitable from an industrial point of view, as a strong adhesion is achieved between the deposited material and the textile substrate [130]. As such, prepared rGO/Ag nanowires fabric can potentially be used for monitoring the external stimuli (e.g., temperature).

Due to the increasing demand for exceptional conductive and electrochemical properties for specific application in wearable textiles, conductive polymers and carbon-based materials have been combined with emerging MXenes [131,132] to fabricate multifunctional e-textiles by spraying. Otherwise, MXenes themselves have already shown their great application in integrated Joule heating, electromagnetic interference shielding and strain sensing when sprayed on fabrics by forming conductive networks (Figure 7c) [127]. Moreover, Mxenes have been incorporated into cellulose-based yarns for textile-based electronic devices [128]. The corresponding morphology of thus produced spray-coated yarns is shown in Figure 7d.

The intensive development of conductive textiles in wearable EMI shielding has led to the combination of high dielectric ZnO NPs and highly conductive rGO and their application on cotton fabrics [129]. The uniformity of the deposited coatings was achieved first by ZnO NPs and then by rGO spraying, the latter having covered the entire ZnO-coated cellulose fibre completely after three cycles of GO (Figure 7e). The cotton fabric prepared in this way achieved the highest overall EMI shielding efficiency of about 99.999% (54.7 bB). In another study, Arumugan et al. [133] fabricated spray-coated fabric solar cells on polyester/cotton fabric also using a layer of ZnO NPs and Ag NW.

Some researchers used a combination of spray coating with other techniques to obtain conductive textiles. Lin et al. [134] proposed a controllable and fast metallic coating technique by combining spray deposition and electroless plating to fabricate a uniform and dense conductive Ag/polyimide fabric with a surface resistance of 0.1 Ω/sq. Zhao et al. [135] developed a sandwich structure using a polyurethane-AgNPs-polyurethane microstructure via the spray coating technique, resulting in a wear-resistant conductive polyester fabric that exhibited excellent conductivity, with surface resistance of 4.95 Ω/sq. Chen et al. [136] employed spray-assisted layer by layer chemical deposition for the preparation of a thin Cu layer with micro-nano structures on carbon fibre fabrics (CFFs) with excellent EMI shielding properties.

## 4. Protection of Conductive Elements on Textiles

While the introduction of electrically conductive elements on textiles increased their usage dramatically in smart wearable applications, the weak interactions between textile substrate and electrically conductive compounds, as well as unstable surface morphology of the textiles, often resulted in poor stability of the conductive layers and limited durability in our daily lives [137]. Conductive metallic layers on textile surfaces are usually chemically unstable and prone to corrosion in water and sweat, affecting the conductivity and flexibility of the material [11]. In addition, due to the external physical-mechanical forces encountered in daily use, metal NPs could peel off from the textiles´ surfaces, causing conductivity decay. To overcome these issues, a protection of the conductive patterns is needed, presenting a significant challenge in the field of E-textiles [138]. A subsequent step of post-deposition or in-situ polymerisation of a protective polymer layer on the top of conductive layer is generally required to improve the durability and stability of the functional conductive layer, which is typically associated with a reduction in conductivity of textiles and a substantial increase in the production cost. Moreover, the protective layer applied over the metallised patterns usually enlarges the thickness of the textile, and thus, influences its flexibility and physical-mechanical properties. It could also block the pores between the fibres and/or yarns, reducing the air permeability and affecting the textile‘s thermal and moisture behaviour [139]. This results in insufficient breathability and comfortability, especially when protective coatings are applied on large surface areas, which is another neglected area when dealing with e-textiles.

Some researchers previously employed an interface polymer layer on the textile surface before metallisation, in order to reduce the inhomogeneity and roughness of textiles, and, thus, enhance the adhesion of the conductive layer [47,115,140,141]. Such intermediate layer allows the under and side protection, and provides electrical insulation (a sealed sandwich like composition) when complemented by the application of an upper-side encapsulation layer on the top [135,141]. The protective layer, also known as a passivation layer, is used to protect the peripheral areas of the active semiconductor surface, which is exposed to the environment. It needs to fulfil several properties, including good adhesion on diverse types of textiles, also during flexing, chemical inertness, corrosion resistant dielectrics (acting as diffusion barriers to water diffusion) and wide bandgap (and, thus, high electrical breakdown strength) [2]. Besides that, protective compounds need to have low environmental impact and a reasonable price.

Significant concern for the commercialization of e-textiles is their wash and wear durability, which is based on the lifetime of electronics and textiles, and ensurement of their projected functional life span. One of the most damaging and destructive processes in the life cycle of an e-textile is the washing process, including water, detergents, high temperatures, mechanical forces, etc., which does not receive enough attention as compared to other performance properties [142]. Nevertheless, several research papers can be found dealing with the improvement of wash and wear durability of conductive layers by application of protective compounds, using coating, laminating, screen-printing or padding processes (Table 2).

As can be seen from Table 2, different authors employed different wash tests and number of washing cycles (from 1 up to 30) to examine wash durability. Kazani et al. [144] screen-printed wearable silver-based conductive antennas on PET and Co/PES (20/80), protected by a thermoplastic polyurethane (TPU) coating, preventing degradation of the conductive area, as well as delamination of the multi-layered material due to washing, making the antennas washable for up to 20 cycles. Ojstršek et al. [2] applied four different protective compounds by screen-printing and padding over the electroless Cu plated PET fabric. The obtained results demonstrated that PDMS, polyurethane and acrylate resins imparted durable protection to Cu against 30 washings, with average electrical resistivity of 12.2 Ω. Jiang et al. [145] applied two commercial solutions of Polyethylene terephthalate-polyurethane (PET-PU) and aqueous acrylate (AC) in eight concentrations over Cu magnetron sputtering PET fabric, obtaining superior protection using 15% of PET-PU as compared to AC. Moreover, with the usage of a cross-cut tape test they assessed the enhanced adhesion strength colourimetrically when the protective layers were applied. Ali et al. [101] demonstrated good wash durability of in-situ deposited Cu particles on cotton fabric protected with an organic-inorganic non-conductive binder, without deterioration of electrical conductivity. Montazer et al. [146] reported the improvement of wash durability of AgNPs on Nylon post-treated with 1,2,3,4-butanetetracarboxylic acid (BTCA) and sodium hypophosphite (SHP).

Although wash durability of e-textiles is one of the main obstacles that stand in the way of a wider market success, there are no standardized methods yet for wash testing of e-textiles, and no protocols to assess the washability vs. electrical conductivity of tested products comparably. The presented insight into current e-textile wash testing methods is limited by the researchers‘ lack of fully disclosed methodology on wash testing. Rotzler et al. [147] underscored the need for e-textile-specific standardisation to overcome the existing lack of comparability between diverse wash processes. They reported that, even though there is no proper standardization for e-textiles yet, in 60% of the reviewed publications, the already used wash standards for testing, which originate from the textile field-testing, are lacking an adequate consideration of the integrated electrically conductive and electronic components in e-textiles. As summarised by [148], just 18 standard test methods published and in development by the CEN, IEC, ASTM, and AATCC technical committees relevant to smart/e-textiles were identified, which is one of the primary restraining factors for industrial growth.

Not just water and detergents, but also skin exudates, mainly chloride ion, low-molecular-weight acids and amino acids in sweat, can oxidise, and possibly dissolve, metal coatings on the surfaces they contact [149], and, consecutively, influence electrical conductivity negatively. Human sweat is typically acidic, but it may become alkaline under high temperatures, or when bacteria are present. Zhang et al. [150] studied the stability of Cu magnetron sputtered/benzotriazole (BTA) protected PES fabric against synthetic perspiration (R22151, ISO 105/E04). The results show that the Cu film treated with 1 g/L of BTA (as a Cu corrosion inhibitor) presented good perspiration durability, with the sheet resistance of 1.15 Ω/sq, and the average of the EMI-SE value was 35 dB. When BTA was increased, both the EMI-SE and conductivity were reduced. In a study performed by Jiang et al. [145], the perspiration durability of two, Cu-sputtered/PP-protected and Cu-sputtered/AC-protected, PES fabrics was tested according to Standard AATCC 15-2013. They found out that the surface of the Cu-coated fabric was eroded severely by sweat, and that PET-PU and AC sealed the Cu film successfully at concentrations of 35% and 40%, respectively, protecting the Cu from the sweat corrosion efficiently.

As already mentioned, protective coatings above a metallic layer could also prevent peeling-off the metal NPs from the textile surface during everyday usage, caused by abrasion forces between textile and skin, textile and textile, and textile and surrounding materials. Thus, it is also very important that protective coatings themselves withstand rubbing, otherwise impaired protection could lead to corrosion of the metal deposit. Although the topic is important for wearable e-textiles, wear durability is neglected by researchers dealing with the protected metallized textiles. Ojstršek et al. [2] studied the abrasion resistance of four protective coatings, i.e., PDMS, polyurethane (PUR), modified acrylate (AR) and epoxy resins (ER) on PES fabric, using the Martindale method and up to 20,000 rubbing cycles by calculation of mass loss. The obtained results showed the superior abrasion resistance of PUR, followed by PDMS. Jiang et al. [146] reported improved dry and wet crocking fastness of 15% PP and 20% AC coatings over Cu-conductive PES fabric, from Grades 2–3 (dry) and 3 (wet) to 4 (dry) and 4–5 (wet), using an AATCC crockmeter according to the AATCC 8-2013 Standard. Zhao et al. [135] tested the abrasion resistance of a polyurethane/silver/polyurethane (PU-Ag-PU) sandwich micro-structure coating layer on PET fabric according to ASTM D3884–2001 up to 1500 test cycles. The wear resistance of the PU-Ag-PU PET fabric was improved significantly as compared to the Ag PET fabric, the weight loss was reduced from 5.57% to 1.28%, with surface resistance reduced from 108 Ω/sq to 82 Ω/sq after 1,500 cycles of abrasion test.

## 5. Conclusions

Rapid on-going development of e-textiles in recent years has been governed by advances in nanotechnology, new material development, innovative approaches in application techniques and miniaturized electronics; these factors, coupled with new solutions in wireless communications and big data analysis, render wearable e-textiles more feasible in a wide range of applications. Metal-based substrates (particle suspensions, ink formulations etc.) for imparting conductivity by way of surface deposition on textiles, are preferred materials of choice to ensure exceptionally high conductivity values (conducting polymers need to be additionally doped to compete with metals in this regard). In the presented review, a variety of metal-based coating techniques for fabrication of electronically conductive textiles are evaluated in a comprehensive manner, encompassing the type of metal and textile, pre-treatment methods, operational parameters and performance, of the most employed metallization techniques (screen-printing, ink-jet printing, electroless plating, electrochemical deposition, CVD, PVD and spray coating), as well as the final application purpose. Since all described metallization techniques come with their own sets of advantages and drawbacks, all of which dictate their selection and usability on a case-by-case basis, it is not possible to single out one method over the others as a general rule, but emphasis can be put on printing methods as manufacturing techniques that manage to meet the majority of processing and application requirements when designing electrically conductive textile materials. The most obvious feature is the continuous nature of printing techniques, allowing for a fast production/deposition of conducting layers over a large surface area, if needed; in addition, already existing, state-of-the-art printing set-ups can be easily adopted for processing of conducting inks (ink-jet printing) or con-ducting coating formulations (screen-printing)—this is an attractive feature, since it enables employing already installed printing devices within textile finishing/production facilities. While ink-jet printing does require effort in designing and preparing conducting inks which possess required rheological and surface tension properties in a fairly narrow window, it must be pointed out that, in comparison to electroless plating and electro-chemical deposition, usage of chemicals and subsequent waste thereof is very low, making it a very environmentally acceptable option. A myriad of published work, as covered in this review, on various materials aspects of e-textile development clearly show that we have reached a stage where numerous options in terms of substrates, pre-treatment, treatment and coating techniques are available, all of which now place a pronounced emphasis on the design and construction stage of e-textiles, as a crucial phase where pro-visions have to be made for the intrinsic flexible nature of textile materials, which in itself presents a challenge towards fabrication of efficiently functioning e-textiles, especially since textile material must also serve as a platform for other components which might be needed and integrated within and coupled with the conductive layers (e.g., microprocessors, sensors, etc.). In this regard, printing techniques offer themselves as profoundly versatile techniques; integration of electronic components onto e-textile can be easily facilitated, since the placement of individual components, their connections to one another and overall assembly of the product can be easily altered, according to the need of the user and the requirements of the device. The printing process allows short response time to new demands and proposed improvements during the e-textile design and testing and integration of additional components is not limited to pre-existing layout of conductive elements (as in the case of fabrics with interwoven conducting yarns), but rather, it enables to build the geometry of components’ assembly in a custom manner, as a function of the optimal placement within the e-textile product.

Despite a massive amount of research work in this field, pertaining to both the basic understanding as well as specific applications of e-textiles, there are still aspects which need to be addressed properly and these are to a large extent of the applied nature, i.e., durability of the fabricated metal layers during usage and, in the case of wearable electronic textiles; cleaning and laundering. Another aspect closely connected to usage and to the consumer perception of the e-textiles is comfort; considerations on the effect of metallization on such material properties as moisture absorption, transport, and thermal behavior is not sufficiently tackled in the reviewed literature, especially when considering work on wearable e-textiles, yet they are essential to the comfort of intended users and also allow unobtrusive usage in different settings. Closely connected with these issues is a lack of unified testing and adequate standards, again pertaining to a large extent to the applied part of the e-textile research. Previous works, which have tackled this issue, focusing on a critical aspect of standardized procedures for the evaluation of conductive coatings’ fastness under different conditions (most prominently, washing and laundering), are discussed in this review; lack of specific standards for e-textiles is critically assessed, especially as it presents a hindrance to an industrial growth in this field.

Lastly, and closely connected to the above issue of scarcity of designated standard for e-textiles, we have highlighted efforts and techniques in the published scientific papers towards ensuring protection and longevity of metal-based conducting coatings. These important issues can get overlooked in several published research works, as one would suspect, especially in those that are not necessarily conducted with a consideration for actual usage or application in mind (which is understandable when tackling more fundamental aspects in the discussed scientific field). However, we have identified several compounds and post- or intermediate-treatments for the protection of conductive layers against oxidation and cracking due to washing, perspiration and rubbing. As mentioned before, here, too, authors are left to their own devices when deciding on the proper conditions for testing and evaluating the efficiency of their respective protection methods.

In order to meet the demands of the ever-expanding market for e-textiles and to reconcile some of the existing issues in designing and constructing of e-textile products, the focus in the future will need be directed toward improving the existing techniques in terms of metal adhesion and durability, by introducing new solutions capable of coping with the advancement of material science and electronics, and thus, enhancing the performance and extension of the e-textile lifetime.

## Figures and Tables

**Table 1:** Overview of the latest research in the field of conductive textiles obtained by the electroless plating process.

Textiles	Catalyst	Electroless Plating Method	Electrical Resistance	Application	Reference
Cotton fabric	Ag or Cu Ag	Cu—modified Cu—conventional	20 or 27 Ω ~62 Ω	EMI shielding, Heating performance	[6]
Polyester fabric	Pd/Sn, Ag NPs or Cu NPs	Cu	0.0095–0.0145 Ω/sq 0.0112–0.0185 Ω/sq 0.0061–0.0079 Ω/sq	E-textiles for wearable devices	[20]
Polyimide fabric	Pd	Ni-W-P	0.08 Ω/sq	EMI shielding	[61]
Polyester fabric	Pd	Cu	0.1–1.5 Ω	Conductive tracks for E-textiles	[2]
Polyamide/polyurethane (67/33) knitted fabric	Pd	Cu	~0.32 Ω/sq ~2 Ω/sq stretched	Stretchable conductor	[21]
Cotton/polyurethane (95/5)	Ag	Ag	~10 Ω ~25 Ω stretched	Strain sensor	[23]
Polyimide fabric	Ag	Ag	0.02 Ω/sq	EMI shielding	[62]
Flax fabric	Ni	Cu-Ni	0.78–1.3 Ω/sq	EMI shielding	[13]
Polyamide/polyurethane (87/13) knitted fabric	Pd/Sn	Ni followed by Au	3.6 ± 0.9 Ω/sq	Light-emitting e-textiles with changeable display patterns	[63]
Cotton fabric	Ag	Ag	233.4 S/cm *	E-textiles in wearable thermal therapy	[64]
Cotton fabric	/	Ag	235.64 ± 3.72 Ω/sq	Supercapacitor electrode	[65]
Polyester textile	Pd in scCO_2_	Ni-P	0.013 Ω	Wearable devices	[66]
Cotton fabric	Ni	Cu	No data	Flexible Li-ion batteries	[67]
Polyamide fabric	Ag	Ag	˂1 Ω/sq	EMI shielding	[68]

* Electrical conductivity.

**Table 2:** Overview of protective coatings on metallized textiles and wash durability testing.

Substrate	Conductive Layer	Protective Coating	Durability Testing	Reference
PET	Cu	PDMS, polyurethane, acrylate and epoxy resins	30 washing cycles ISO 105-C06	[2]
Escalade and Lagonda	Ag	Polyurethane	10 domestic washes/40 °C/1000 rpm	[47]
PET, cotton	Nano-Ag flakes	Aliphatic polyurethane acrylate	10 washing cycles	[46]
Modified elastic polypropylene	MXene	Polydimethylsiloxane (PDMS)		[143]
Cotton/PET (20/80) and PET	Ag	Thermoplastic polyurethane	20 washing cycles ISO 6330:2000	[144]
PET	Cu	Polyester-polyurethane and aqueous acrylate	Washing fastness AATCC 61	[145]
PET/cotton (65/35)	Ag	UV-curable polyurethane acrylate paste	Soaking for 24 h	[141]
Cotton	Cu	Organic-inorganic binder system	1 washing cycle ISO 105-C01	[101]
Nylon	Ag	1,2,3,4-butanetetracarboxylic acid (BTCA) and sodium hypophosphite (SHP)	20 washing cycles ISO 105-C01	[146]
